# In vivo experimental intervertebral disc degeneration induced by bleomycin in the rhesus monkey

**DOI:** 10.1186/1471-2474-15-340

**Published:** 2014-10-09

**Authors:** Fuxin Wei, Rui Zhong, Zhiyu Zhou, Le Wang, Ximin Pan, Shangbin Cui, Xuenong Zou, Manman Gao, Haixing Sun, Wenfang Chen, Shaoyu Liu

**Affiliations:** Department of Spine Surgery, the First Affiliated Hospital and Orthopedic Research Institute of Sun Yat-sen University, Guangzhou, China; The medical school of Shenzhen University, Shenzhen, China; Department of Radiology, the First Affiliated Hospital of Sun Yat-sen University, Guangzhou, China; Department of Pathology, the First Affiliated Hospital of Sun Yat-sen University, Guangzhou, China

**Keywords:** Intervertebral disc degeneration, Bleomycin, Animal model, Magnetic resonance imaging, Rhesus monkey

## Abstract

**Background:**

Recently, biological therapies for early intervention of degenerative disc disease have been introduced and developed; however, a functional animal model that mimics slowly progressive disc degeneration of humans does not exist. The objective of this study was to establish a slowly progressive and reproducible intervertebral disc (IVD) degeneration model.

**Methods:**

The subchondral bone adjacent to the lumbar IVDs (L3/4 and L5/6) of ten rhesus monkeys was randomly injected with 4 ml bleomycin solution (1.5 mg/ml), or 4 ml phosphate buffer saline (PBS) per segment as control, respectively. The degenerative process was investigated by using radiography and T1ρ MR imaging at 1, 3, 6, 9, 12 and 15 months postoperatively. Histological scoring, Sulfated Glycosaminoglycans (GAGs) analysis and real-time PCR were performed at 15 months. The correlation between histological score, GAGs and T1ρ values were also analyzed.

**Results:**

The results showed that the mean T1ρ values of nucleus pulposus (NP) and annulus fibrosus (AF) in the bleomycin group significantly decreased after 3 and 6 months respectively, followed by slowly decrease until at 15 months. At 15 months, the histological scores was significantly higher, and the GAGs of NP was significantly lower in the bleomycin group, compared with the control group (*P* < 0.05). The results of real-time PCR revealed a significant increase in matrix metalloprotease (MMP)-3, A disintegrin and metalloproteinase with thrombospondin motifs (ADAMTS)-5, tumor necrosis factor α, interleukin-1β, interleukin-6 expressions, transforming growth factor (TGF-β1) and marked reduction in aggrecan, type II collagen, von willebrand factor (vWF) expressions at the mRNA levels in the bleomycin group. Spearman correlation analysis showed a strong positive correlation between GAGs and T1ρ values of NP (r =0.740, *P* < 0.01), and a significant inverse correlation between histological score and T1ρ values of NP and AF (r = -0.761, r = -0.729, respectively, *P* < 0.01).

**Conclusions:**

Injection of bleomycin into the subchondral bone adjacent to the lumbar IVDs of rhesus monkeys can results in mild, slowly progressive disc degeneration, which mimics the onset of human disc degeneration. T1ρ MR imaging is an effective and noninvasive technique for assessment of early stage disc degeneration.

**Electronic supplementary material:**

The online version of this article (doi:10.1186/1471-2474-15-340) contains supplementary material, which is available to authorized users.

## Background

Intervertebral disc (IVD) degeneration,which is an epidemic human condition with a major socioeconomic impact, is believed to be a main cause of low back pain [1]. However, a clear understanding of the basic mechanisms of disease pathogenesis and specific therapeutic agents is still limited [2]. An experimental animal model of human IVD degeneration is needed. It is well known that IVD is the largest avascular structure in the human body that has limit the capacity for regeneration. Although there are several animal models of experimental disc degeneration that have been developed, which including spontaneous degeneration models [3], annulus fibrosus injury models [4], and chemically induced models et al. [5], these methods could not model the slowly ischemic and progressive disc degeneration of human beings effectively. Although the needle puncture methods have been recently developed and used to induce disc degeneration models to test the effectiveness of new therapies for reversing IVD degeneration [4], this method induces to IVD degeneration through direct injury, which is not easy to control the degenerative process. Furthermore, these models are not suitable for the studies related to biological treatment of IVD degeneration to some extent.

The nutritional pathways that into the nucleus pulposus of human intervertebral discs are mainly by diffusion through the central portion of the end-plate from these marrow space cartilage contacts and diffusion through the annulus fibrosus from the surrounding vessels [6]. An alternation of the nutrient pathway is considered as one of the main causes of IVD degeneration [7]. Bleomycin, which have been used as sclerosing agents, has proved to be safe and effective in the treatment of vascular malformations of the head and neck [8, 9]. Intralesionally injected bleomycin brings the drug into direct contact with the endothelial lining and destroys the endothelial cells, resulting in sclerostenosis of the lumen and leading to narrowing or occlusion of the vessels [10].

The purpose of this study was to establish an early ischemic, progressive, and reproducible IVD degeneration model induced by injection of bleomycin into subchondral bone adjacent to the intervertebral disc of rhesus monkeys to provide a basis for further in vivo studies on IVD degeneration. Combined with histology, real-time PCR and Sulfated Glycosaminoglycans (GAGs) assessment, the T1ρ magnetic resonance imaging (MRI) technique [11, 12], which has been reported strongly correlated with proteoglycan content were performed to evaluate the progression of IVD degeneration.

## Methods

### Animals

Ten rhesus monkeys (six females and four males), with a mean age of 5.32 ± 0.47 years old (range, 5–7 years), which can be converted to equivalent human age by using a 1:3.5 ratio [13], and a mean body weight of 6.83 ± 0.67 kg (range, 5.2-7.5 kg) were used in this study. All of the animals were provided by the center of Guangdong Landao Biotechnology in Guangzhou of China, and exclude spinal deformity by radiological examination. The study protocol was reviewed and approved by the institutional review board and ethics committee of the First Affiliated Hospital of our university (No. 2010–204). The animal study was also approved by the institutional review board and animal care committee of the First Affiliated Hospital of our university (2013A-204).

### Animal groups

In this study, a single concentration of 1.5 mg/ml bleomycin in phosphate buffered saline (PBS) was performed. Unlike human beings, there are 7 intervertebral discs (IVDs) in rhesus monkeys. The subchondral bone adjacent to the 2 lumbar IVDs (L3/4 and L5/6) were randomly injected with 4 ml bleomycin solution (Bleomycin, n = 10), or 4 ml PBS (Control, n = 10), respectively. The degenerative process was investigated by using radiography, T1ρ MR imaging for 15 months.

### Experimental surgery

All the experimental surgeries were performed in the orthopedic research institute of Sun Yat-sen University. Intramuscular injections of 10 mg/kg ketamine and 0.5 mg/kg midazolam were used for anesthesia of the monkeys, followed by 0.3 mg/kg midazolam and 4 μg fentanyl per hour. Under sterile surgical conditions, the vertebral bodies from L3 to L6 were exposed by using a left retroperitoneal approach. After clearly identified, the subchondral bone adjacent to the IVDs of L3-4 and L5-6 was performed according to the preoperative design. To induce the subendplate ischemic model, a hole 1.5 mm in diameter and 15 mm in depth was drilled in the middle of the intervertebral body approximately 1.5 mm above and below the disk, followed by slow injection of 2.0 ml bleomycin (1.5 mg/ml, Tianjin Taihe Pharmaceutical, Tianjin, People’s Republic of China) per hole for the belomycin group, and the same volume of PBS for the control group, respectively. Then, each hole was sealed with bone wax.

Before, during, and after operation, the monkeys were given 80 mg per kilogram of ceftriaxone sodium (Baiyun Pharmaceuticals, Guangzhou, China) subcutaneously. After operation, the monkeys were housed individually with free access to water. The rhesus monkeys tolerated general anesthesia well and no mortalities from complications caused by anesthesia or infections were found among the animals. Weight, food intake, and sleeping habits were recorded.

### IVD height measurement

**Figure 1:**
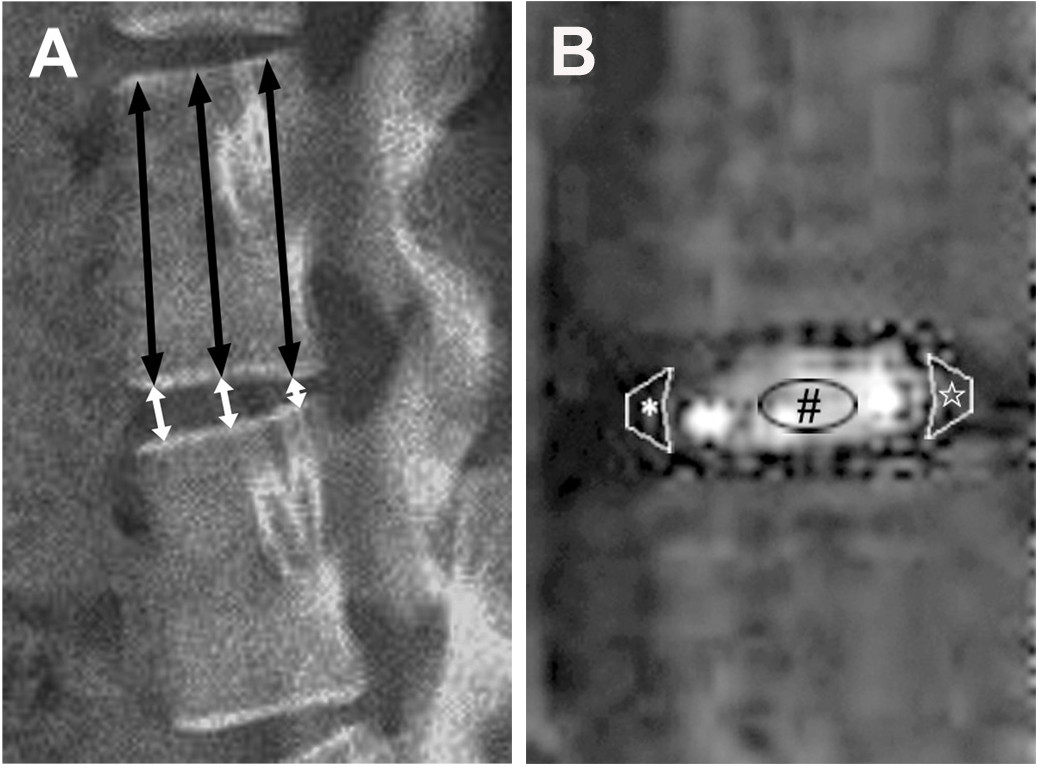
**Radiography and T1ρ-weighted images of a part of the lumbar spine of rhesus monkeys. (A)** The Disc Height Index was calculated by dividing the average disc height (white arrows) by the average vertebral body height (blasck arrows). **(B)** An example of placement of ROIs over NP(#), anterior AF(*) and posterior AF(☆) was showed in the IVD respectively. ROIs: regions of interest; NP: nucleus pulposus; AF: annulus fibrosus; IVD: Intervertebral disc.

The lateral views of roentgenograms for the lumbar spine were acquired for all the monkeys by using the Digital Diagnost VM (Philips, Best, the Netherlands), a multipurpose single-detector digital radiography system. The average IVD height was measured manually by an orthopedic researcher (R. Z.) who was blinded to both the injection solution used (PBS/Belomycin) and the follow-up period. The average IVD height was calculated by averaging 3 measurements (Figure 1A). The average IVD height was obtained concordantly. Subsequently, the average IVD height was divided by the average caudal vertebral height,resulting in a value called the disc height index (DHI) leveling out interanimal size difference [14]. Changes in the DHI were expressed as %DHI and normalized to the measured preoperative DHI (%DHI = postoperative DHI/preoperative DHI × 100%) [15].

### MR imaging analysis

Each animal underwent MRI on a 1.5-T MR imager (Achieva, Philips Healthcare, Best, The Netherlands) with a supine position on a spine-array coil (5 × 11 inches). The T1ρ-weighted images were acquired by using spin-lock pulses followed by a spin-echo acquisition, with time of spin-lock of 2, 15, 30, and 45 msec and spin-lock frequency of 250 Hz. Using the Siswin software, we mapped these data and exported the data to mapping images. Article by Zhou et al. contains details of the T1ρ sequence [16]. The T1ρ values were calculated on the basis of selection of the regions of interest (ROIs) [16], which were manually drawn over the T1ρ maps of the discs by a radiologists (X.P.) with more than 5 years’ experience reading MRI images. ROIs included nucleus pulposus (NP), anterior annulus fibrosus (AF) and posterior AF (Figure 1B). Values of anterior and posterior AF were averaged as the value for AF. To evaluate ROIs in a standardized and reproducible way, we decided to measure oval ROIs (0.1 cm^2^ on NP, 0.05 cm^2^ on AF). The average of three mid-sagittal values from one disk was used for the statistical analysis.

### Tissue harvesting

Disc tissues, which involved adjacent endplates, were harvested from all the rhesus monkeys under anesthesia with intramuscular injections of 10 mg/kg ketamine and 0.5 mg/kg midazolam, at 15 months after surgery. All the specimens were dissected into two halves in the sagittal plane, one half was fixed for histology analysis, and the other half was sub-dissected into another two halves, which were used for messenger RNA (mRNA) analysis and Sulfated Glycosaminoglycans (GAGs) assessment.

### Histology evaluation and histological scores

The specimens were fixed with 4% paraformaldehyde for 24 hours at 4°C, then transferred to a sealed vial containing a solution of 70% ethanol and decalcifying agent for 30 days. After washed with water, the specimens were sequentially dehydrated, split down the mid-sagittal plane, embedded in paraffin for histology sectioning. Serial sections were cut in the transverse plane at 8 μm with a microtome (Microtome International, Waldorf, Germany), and then stained with hematoxylin and eosin (H&E), masson trichrome or safranin O staining for light microscopic examination.

The histological changes were qualitatively analyzed by a pathologist (W.CH.) using a digital image analysis system (Nikon Eclipse Ti; Nikon, Tokyo, Japan) who was blinded to the different treatments between groups, according to the classification scale described by Masuda et al. [4]. The criteria are summarized in Table 1. The histological score is the sum of the scores of the 3 individual parameters and ranged from 3 to 9, where normal is 1point for each of the 3 categories listed above, for a total of 3 points. A total of 9 points is representative of severe degeneration.

**Table 1:** **Degeneration histologic grading scale**

**I Anulus fibrosus**	
Grade:	
1	Normal, pattern of fibrocartilage lamellae without ruptured fibers and without a serpentine appearance anywhere within the anulus
2	Ruptured or serpentine patterned fibers in less than 30% of the anulus
3	Ruptured or serpentine patterned fibers in more than 30% of the anulus
**II Border between the anulus fibrosus and nucleus pulposus**	
Grade:	
1	Normal
2	Minimally interrupted
3	Moderate/severe interruption
**III Cellularity of the nucleus pulposus**	
Grade:	
1	Normal cellularity with large vacuoles in the gelatinous structure of the matrix
2	Slight decrease in the number of cells and fewer vacuoles
3	Moderate/severe decrease (>50%) in the number of cells and no vacuoles

### Sulfated Glycosaminoglycans (GAGs) assay

NP from the other half samples were digested using papain at 65°C for 2 hours. GAG content was calculated in duplicate by dimethylmethylene blue (DMMB) procedure [17], using chondroitin-4-sulfate (Sigma-27042) as a standard. The DNA concentration of each sample was measured using the PicoGreen assay (Molecular Probe) and used for normalizing the GAG values.

### Real-time PCR analysis

Total RNA were extracted from the specimens using Trizol reagent (Ambion, Carllsbad, CA, USA), followed by the RNeasy Mini Kit (Qiagen Inc., Duesseldorf, Germany). Reverse transcription was performed at 42°C for 50 minutes using the SuperScript First-strand Synthesis Kit (TOYOBO, Biotech, Co., Ltd., Shanghai, China). Aggrecan, type Icollagen (Col1α1), type IIcollagen (Col2α1), Matrix metalloproteinases (MMP)-3, A disintegrin and metalloproteinase with thrombospondin motifs (ADAMTS)-5, tumor necrosis factor α (TNFα), interleukin-1β (IL-1β), interleukin-6 expressions (IL-6), caveolin-1, transforming growth factor-β1 (TGF-β1) and glyceraldehyde 3-phosphate dehydrogenase (GAPDH) gene expression of the intervertebral discs were quantified by real-time PCR using CFX96 Real-Time System (Bio-Rad, Herculus, CA, USA). The sequence of all the primers used for real-time PCR are shown in Table 2. With a serially-diluted cDNA sample mixture, a positive standard curve for each primer was obtained for real-time PCR. Quantitations of gene expression of aggrecan, Col1α1, Col2α1, MMP-3, ADAMTS-5, TNFα, IL-1β, IL-6, caveolin-1 and TGF-β1 in the intervertebral discs were calculated using standard curves and normalized to GAPDH in each specimen. In order to evaluate the number of capillaries in the cartilage endplate, the quantitation of gene expression of von willebrand factor (vWF) in the bone endplates was also calculated using the same method as mentioned above. The experiments were repeated at least twice for enhanced accuracy.

**Table 2:** **Sequences of primers used in the real-time PCR**

Name	GeneBank accession No.		Sequence (5′-3′)
GAPDH	XM_001091567.2	Forward	ACAATCCCATCACCATCTCGC
		Reverse	GACCCTTTTGGCCTCCTCATC
Aggrecan	XM_002804944.1	Forward	ACTCGCTGAGTGTCAGCATC
		Reverse	ACACACGGCTCCACTTGATT
Col1α1	XM_001096194.2	Forward	CCAGCCGCAAAGAGTCTACA
		Reverse	TGGTGGGATGTCTTCGTCTTG
Col2α1	XM_001100559.2	Forward	GTGTCAGGGCCAGGATGTC
		Reverse	AGGGGCACCTTTTTCACCTT
MMP-3	XM_001098400.2	Forward	GGCGCAAATCTCTCAGGAAG
		Reverse	GGCCCAGAACTGATTTCCTTT
ADAMTS-5	XM_001103439.2	Forward	GTGGCTCACGAAATCGGACA
		Reverse	GGTGGCTGAAGTGCATTTGG
vWF	NM_001243086.1	Forward	GAGGGTGGTTGGTGGATGTC
		Reverse	CTAGGCCATGCTCCTAGCTG
TNF-α	NM_001047149	Forward	CCCCAAGGACCCCTCTCTAA
		Reverse	GGGTTTGCTACAACATGGGC
IL-1β	NM_001042756	Forward	GACGTCGATGGCCCTAAACA
		Reverse	AAGCCCTCGTTGTAGTGCTC
IL-6	NM_001042733	Forward	GGTACATCCTCGACGGCATC
		Reverse	CCAGGCAAGTGTCCTCATTG
Caveolin 1	NM_001168614	Forward	ACGTAGACTCGGAGGGACAT
		Reverse	AGCGATGGTGATTCCCCAAG
TGF β1	XM_001100842	Forward	GGGCTACCATGCCAACTTCT
		Reverse	CCAGGACCTTGCTGTACTGT

### Statistical analysis

SPSS 16.0 software (SPSS Inc., Chicago, IL) was used for univariate analysis of variance. The data within groups were analyzed using One-way Analysis of Variance (ANOVA) and Fisher’s protected least significant difference test. The data between groups were compared by using paired *t* test. The correlation between the %DHI, histological score, GAGs content, and T1ρ values of NP and AF were assessed by the Spearmann rank correlation test. Data are presented as the mean ± standard deviation. Statistical significance was indicated at *P* < 0.05.

## Results

No postoperative morbidity or mortality was recorded. All animals recovered uneventfully after surgery and quickly resumed normal activities in the cage. None of these animals showed any remarkable change in weight, eating patterns, or sleeping habits.

### Intervertebral disc height

**Figure 2:**
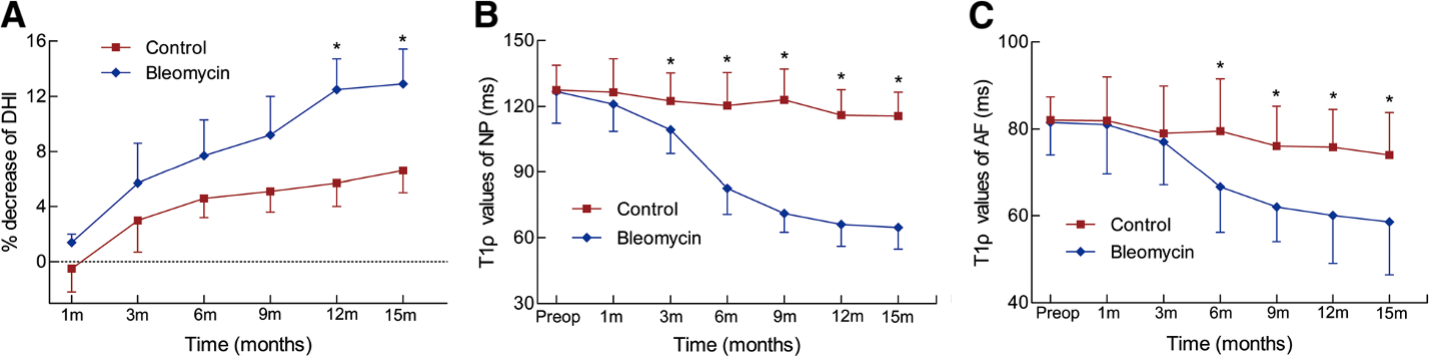
**Mean %DHI, T1ρ values of NP and AF at each time point. (A)** A significant decrease of %DHI was observed at 12 months postoperatively in the bleomycin group (*P < 0.05). **(B)** A significant decrease in the T1ρ values of NP was observed at 3 months postoperatively in the bleomycin group (*P < 0.05), followed by a rapid decrease during the second 3 months. **(C)** A significant decrease in the T1ρ values of AF was observed at 6 months postoperatively in the bleomycin group (*P < 0.05), followed by slowly decrease. DHI: Disc height index; NP: nucleus pulposus; AF: annulus fibrosus.

In the control groups, the DHI changes were not significant at any time point examined. In contrast, the DHI in the bleomycin group progressively decreased over time (Figure 2A), reaching significance when compared with that in the control group after 12 months (*P* = 0.02), and was followed by slowly declining. After 15 months, the DHI decreased by 12.9%, which was not significant, compared with the DHI at the 12-month time point (*P* = 0.76).

### Histological findings and GAGs assay

**Figure 3:**
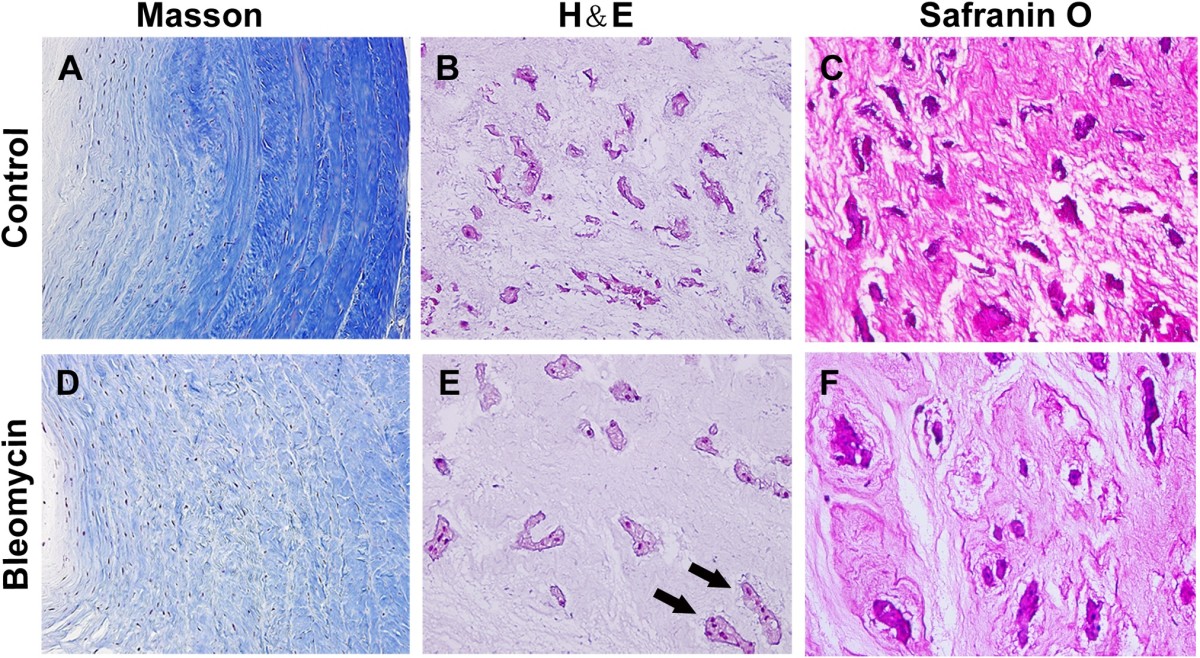
**Hematoxylin and eosin (H&E), masson trichrome and safranin O staining for NP and AF. (A)-(C)** The annulus fibrosus showed normal organization of fibrocartilage lamellae, and the nucleus pulposus contained abundant cells and sounded by large zones of acellular matrix in the control group. **(D)-(F)** In the bleomycin group, the nucleus pulposus comprised relatively few cells and less proteoglycans, combined with less organized fibrocartilage lamellae and a wavy arrangement of collagen fibers, relative to the control group. Black arrows indicate notochordal cells based on morphology.

The intervertebral discs in the control group appeared normal. The annulus fibrosus showed normal organization of fibrocartilage lamellae (Figure 3A), and the nucleus pulposus contained abundant cells and sounded by large zones of acellular matrix (Figure 3B,C). In the bleomycin group, the discs showed degenerative changes, where the nucleus pulposus comprised relatively few cells (Figure 3E) and less proteoglycans (Figure 3F), relative to the control group. The annulus fibrosus showed less organized fibrocartilage lamellae, and the collagen fibers formed a wavy arrangement (Figure 3D) in the bleomycin group.

**Figure 4:**
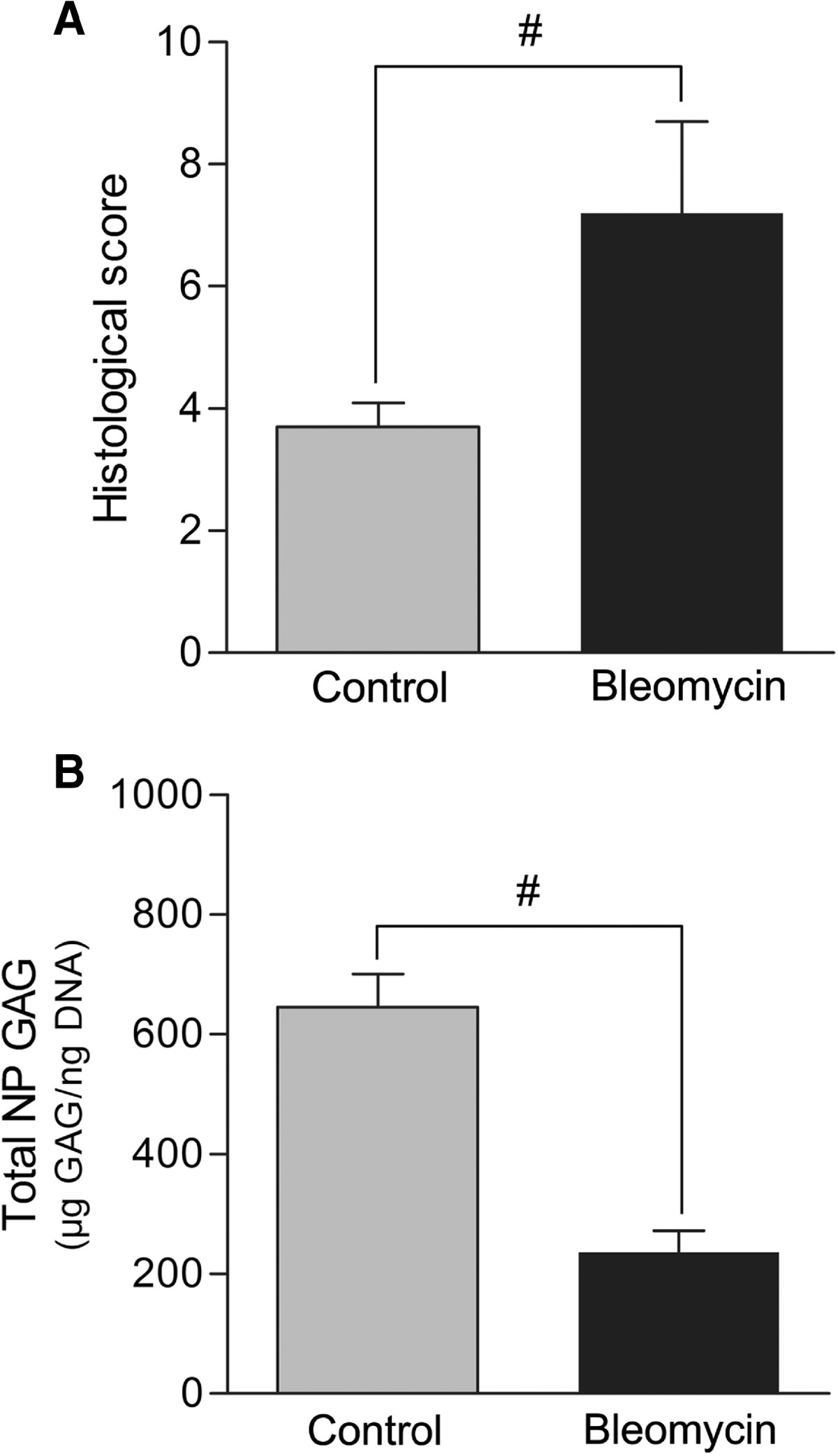
**Bar graphs illustrating histological score and total NP GAGs between groups. (A)** The histological score of the discs in the bleomycin group was significantly higher than the control groups (^#^ *P* < 0.01). **(B)** The total GAG of NP in the bleomycin group decreased significantly compared with the control group (^#^ *P* < 0.01). GAGs: Glycosaminoglycans; NP: nucleus pulposus.

The histological score of the discs in the bleomycin group was significantly higher than the control groups (*P* < 0.01, Figure 4A). The total GAG of NP in the bleomycin group decreased significantly compared with the control group (*P* < 0.01, Figure 4B).

### MRI

**Figure 5:**
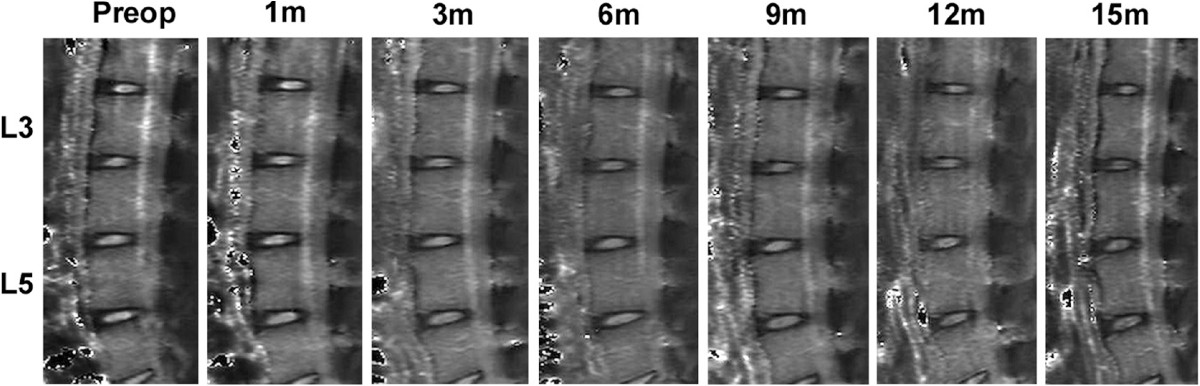
**The representative T1ρ maps of lumbar spine in an rhesus monkey model.** Bleomycin and PBS solution was injected at L3-4 and L5-6 respectively. Preop: Preoperatively.

The MRI images showed degenerative signs in IVDs injected with bleomycin (Figure 5). There were no significant differences in T1ρ values of NP and AF in the control group at any time point. In the belomycin group, the T1ρ values of NP and AF slowly declined after operation. After 3 months, the decrease in NP was significant (*P* = 0.02, Figure 2B). However, the decrease in AF did not show any significant difference, until 6 months after operation (Figure 2C). Interestingly, the T1ρ values of NP showed a rapid decrease during the second 3 months from 109.3 msec ±10.8 to 82.6 msec ±11.9 (*P* = 0.003), in the bleomycin group, and then decreased slowly. After 12 months, both decrease of T1ρ values in NP and AF tended to be stable.

**Figure 6:**
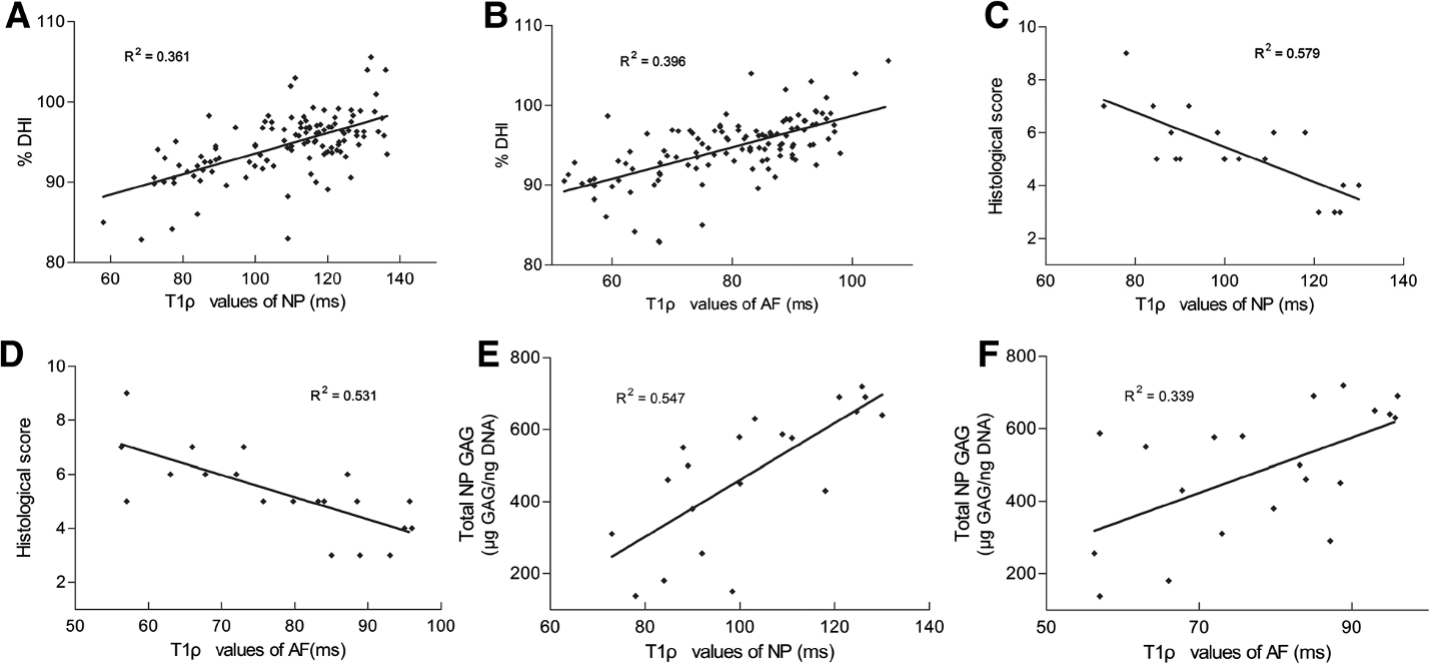
**Correlations of T1ρ values to %DHI, histological score, and total NP GAGs. (A)-(B)** A mild correlation was observed between %DHI and T1ρ values of NP and AF(*P* < 0.01). **(C)-(D)** A strong inverse correlation was found between histological score and T1ρ values of NP and AF(*P* < 0.01). **(E)-(F)** The total NP GAGs change correlated significantly with the T1ρ values of NP(*P* < 0.01), wheares the T1ρ values of AF did so more weakly than that of NP(*P* < 0.05).

The correlation of T1ρ values to %DHI, histological score, or GAGs content was assessed by the Spearman rank correlation test. Although both of the T1ρ values of NP and AF negatively correlated with the histological score, and positively correlated with the total NP GAGs significantly (*P* < 0.05, Figure 6, Table 3), the T1ρ values of AF did so more weakly than that of NP. However, the positive correlation between the T1ρ values of AF and the %DHI was much stronger than that of NP (Table 3).

**Table 3:** **The correlation among disc height index, histological score, total NP GAGs and T1ρ values (Sperman rank correlation coefficient, rho)**

	% DHI	Histological score	Total NP GAGs
T1ρ values of NP	0.601	-0.761	0.740
	*P* < 0.0001	*P* < 0.0001	*P* < 0.0001
T1ρ values of AF	0.630	-0.729	0.582
	*P* < 0.0001	*P* = 0.0003	*P* = 0.02

NP = nucleus pulposus; AF = annulus fibrosus; % DHI = change in disc height index.

GAGs = glycosaminoglycans.

### Real-time PCR

**Figure 7:**
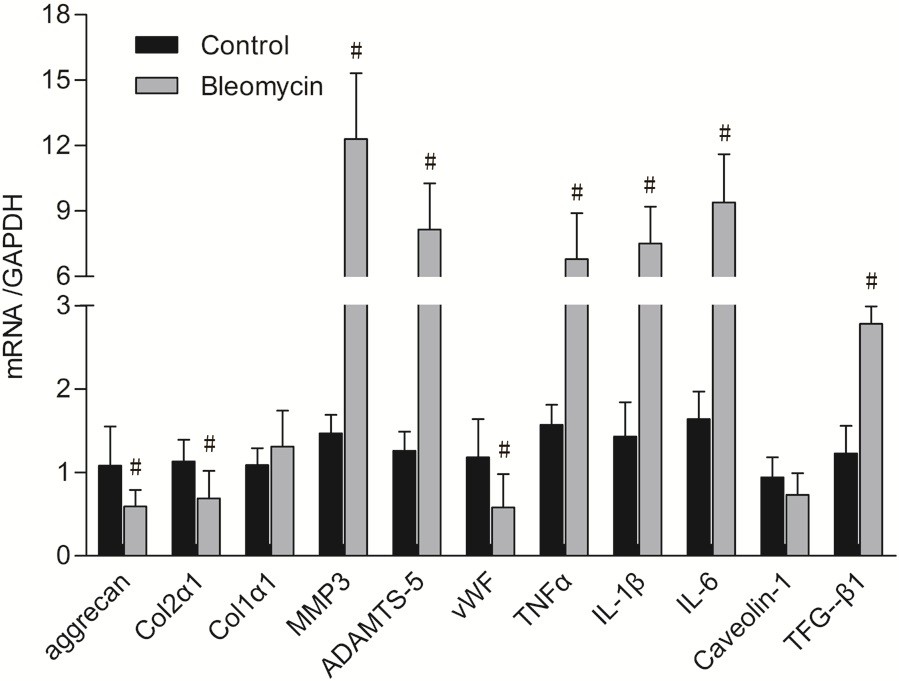
**mRNA expression of genes at 15 months postoperatively.** This graph showed a lower expression of aggrecan, Col2α1 and caveolin-1, and a significant upregulation of MMP-3, ADAMTS-5, TNF-α, IL-1β, IL-6 and TGF-β1 in the bleomycin group as compared with the control group (P < 0.05). The expression of vWF in the bleomycin group significantly decreased in comparison with the control group (P < 0.05). ^#^P < 0.05 *vs* the control group.

The expression of Aggrecan and Col2α1 were significantly lower, and the expression of MMP-3, ADAMTS-5, TNF-α, IL-1β, IL-6 and TGF-β1 were significantly upregulated in the bleomycin group as compared with the control group (*P* < 0.05, Figure 7). The expression of vWF in the bleomycin group significantly decreased in comparison with the control groups (*P* < 0.05, Figure 7). However, there were no significant differences in the expression of Col1α1 and caveolin-1 between groups (*P* > 0.05, Figure 7).

## Discussion

It has been generally recognized that degenerative disc disease is the most common cause of low back pain. Recently, biological therapies for early intervention of degenerative disc disease have been introduced and developed, including direct delivery of specific growth factors, plate-rich plasma, adenovirus particles or autologous cells, into the disc [18–20]. Thus, it is essential to establish a suitable disc degenerative model that could better model the human disease process to evaluate the novel treatment options. However, to our known, there are no particular models currently parallel the complex nature of human disc degeneration [17, 21]. In the present study, we designed a new method of inducing an IVD degeneration model in the rhesus monkey and have demonstrated that it could induce slowly progressive and mild disc degeneration, which mimicked the onset of human disc degeneration.

It is known that the nutrient exchange for disc cells is mostly from the capillaries of the subchondral plate of the vertebral body, across the layers of hyaline cartilage that constitute the endplate [22]. Disorders that affect the blood supply to the endplate are significantly associated with disc degeneration and back pain [23]. In our study, we injected bleomycin,which could produce a “devascular effect” [8, 9], into the subchondral bone adjacent to the IVDs of rhesus monkeys. The results of quantitative T1ρ MR imaging showed that the T1ρ values of nucleus pulposus and annulus fibrosus decreased slowly after surgery, compared with the control group. The degenerative signs of this model developed slower, compared with other animal models, such as needle puncture models or chemically induced models, which were reported to degenerate significantly within 2 to 4 weeks [21]. However, as disc degeneration develops in several decades in humans, the slow development of the degeneration in the rhesus model is relative. In this study, another interesting finding was that, the results showed a great decrease in T1ρ values of NP in the second 3 months after surgery. This was similar to the degenerative process of human subjects, which had also showed a rapid decrease in T1ρ values between Pfirrman gradesIIand III reported by Zhou et al. [16]. This, to some extent, suggested that the degenerative process of this model may be a better surrogate used for investigation of early stage disc degeneration and regeneration studies.

The histomorphological findings and GAGs assay showed that the GAGs in nucleus pulposus decreased significantly in the bleomycin group. These changes were consistent with the previous studies showing loss of proteoglycans during the initial phase of disc degeneration [24]. In human discs, the notochordal cells (NCs) are lost during adolescence, which is also when discs begin to show degenerative signs [25]. Although the cell population significantly decreased in nucleus pulposus of this degenerative model of rhesus monkeys, notochordal cells were still observed. This may be related to the age of the rhesus monkeys. Although this limits relevance to humans, these changes in the degenerative model of this study confirmed that injection of bleomycin into the subchondral bone adjacent IVD of rhesus monkeys could induce early disc degeneration, which showed some similar characteristic features to the complex processes of disc degeneration in human, such as disc height loss, significant decrease of cell population and glycosaminoglycans in the nucleus pulposus, and less organized fibrocartilage lamellae in the annulus fibrosus, et al.

As previously reported, the histological scale and radiographic findings for assessing IVD degeneration showed a consistency to MRI evaluation [4]. In this study, with Sperman rank correlation coefficient of -0.761 and -0.729 (*P* < 0.01), the correlation between the histological scores and the T1ρ values of NP and AF are significant. Although the correlation between the %DHI and the T1ρ values of NP and AF are also significant, but not so strong. This may be due to the different sensitivity of histological and disc height analyses in response to disc degeneration. Early disc degeneration with a slight loss of nucleus pulposus that may be easily detected by histology, may not show significant differences in the disc height change, which is largely affected by the structural changes. It has been reported that T1ρ MRI imaging is strongly correlated with proteoglycan content [11, 12]. This is also observed in our study, which showed strong correlation between the total NP GAGs and the T1ρ values of NP and AF.

It has been revealed that proteoglycans has the ability to bind to collagen, growth factors, and other matrix components [26]. In this study, it has been shown that the collagenIIexpression in the discs of bleomycin group was reduced compared with that in the control group, which was consistent with the theory that proteoglycans can bind to collagens. The degenerative changes in the IVDs, such as composition of extracellular matrix (ECM), loss of disc cells, proteoglycan and water content, have been suggested to be the consequence of an up-regulation of catabolic MMPs and two major aggrecanases, ADAMTS-4 and 5 [27, 28]. Consistent with the previous reports [27, 28], we found that mRNA expression of MMP-3 and ADAMTS-5 were significantly increased in the discs of bleomycin group compared to the control group. This may be due to the abnormal nutrient transport between the IVD and endplates due to the “devascular effect” of bleomycin, which was confirmed by the mRNA expression of vWF in the bone endplates.

It has been reported that the cytokines TNF-α, IL-1β, IL-6 and IL-8 increased significantly in aged and/or degenerated IVDs [29–31]. In this study, there were significant increase of the gene expression of TNF-α, IL-1β and IL-6 in the IVD of bleomycin group. It has been confirmed that hypoxia could increases oxidative stress in several cell types [32, 33], including the notochordal intervertebral disc cells [34]. In this study, the nutrition exchange of the IVD was disturbed by injection of bleomycin into the subchondral bone adjacent to the IVDs of rhesus monkeys. This could disturb the oxygen supply to the IVDs, and then induced increasing oxidative stress, which might account for the increasing gene expression of inflammatory cytokines in the IVDs.

TGF-β is a multifunctional regulator of cellular proliferation, differentiation, and extracellular matrix (ECM) production. Thompson et al. [35] reported the anabolic effect of TGF-β on proteoglycans (PGs) synthesis in canine disc cells. Lee et al. [36] found that the level of TGF-β was higher in patients with disc degenerative disease (DDD). However, it has also been reported that overexpression of TGF-β showed deleterious effect on degenerated cartilage tissue *via* increase of aggrecanase-1 and MMP-13 [37, 38]. In this study, the gene expression of TGF-β was more higher in the degenerative discs, which was consistent with the previous reports [35, 36]. Caveolin-1, which is a scaffold protein of caveolae, is elevated in degenerative discs and has been proposed to play a prominent role in the pathogenesis of IVD degeneration [39]. However, Smolder et al. [40] found that IVD degeneration involved significant down-regulation in caveolin-1. In this study, the gene expression of caveolin-1 was down-regulated in the degenerative discs. This may be due to the effect of bleomycin, which can lead to caveolin-1 down-regulation in fibrosing lung [41]. No matter what reason it is, however, further studies are warranted to evaluate the role of caveolin-1 in disc degenerative disease.

In our study, rhesus monkeys, higher in the phylogenetic tree, were used because of the similarities of their anatomic and physiological characteristics, and IVD anatomy similar to that of humans [42]. Thus, this ischemic degenerative model in the present study could better simulate the IVD degeneration of humans. However, this study also has some limitations. We did not perform the histological evaluation during the follow up period, due to the high cost and limited number of animals. Another limitation was that we did not perform the biomechanical evaluation, such as hydrostatic pressure in the degenerative discs. Additional studies are warranted to further evaluate the mechanism and characters of disc degeneration induced by bleomycin.

## Conclusions

This current study demonstrate that the injection of bleomycin into the subchondral bone adjacent to the lumbar IVDs of rhesus monkeys can induce slowly progressive and mild disc degeneration, which mimics the onset of human disc degeneration. T1ρ MR imaging is an effective and noninvasive technique for assessment of disc degeneration. The degeneration model is suitable for disc degeneration and regeneration studies. Further studies to fully establish this model, however, are needed.

## Authors’ original submitted files for images

Below are the links to the authors’ original submitted files for images. Authors’ original file for figure 1 Authors’ original file for figure 2 Authors’ original file for figure 3 Authors’ original file for figure 4 Authors’ original file for figure 5 Authors’ original file for figure 6 Authors’ original file for figure 7

## Abbreviations

IVD: Intervertebral disc
GAGs: Glycosaminoglycans
DHI: Disc height index
ROIs: Regions of interest
NP: Nucleus pulposus
AF: Annulus fibrosus
H&E: Hematoxylin and eosin
DMMB: Dimethylmethylene blue
MMP: Matrix metalloproteinases
ADAMTS: A disintegrin and metalloproteinase with thrombospondin motifs
GAPDH: Glyceraldehyde 3-phosphate dehydrogenase
Col1: Type Icollagen
Col2: TypeIIcollagen
vWF: Von willebrand factor
TGF: Transforming growth factor
DDD: Disc degenerative disease.
