# Supplementary figures and images for: In vivo experimental intervertebral disc degeneration induced by bleomycin in the rhesus monkey
Source: BMC Musculoskelet Disord. 2014 Oct 9;15:340. doi: 10.1186/1471-2474-15-340 (PMC4210630; doi:10.1186/1471-2474-15-340)

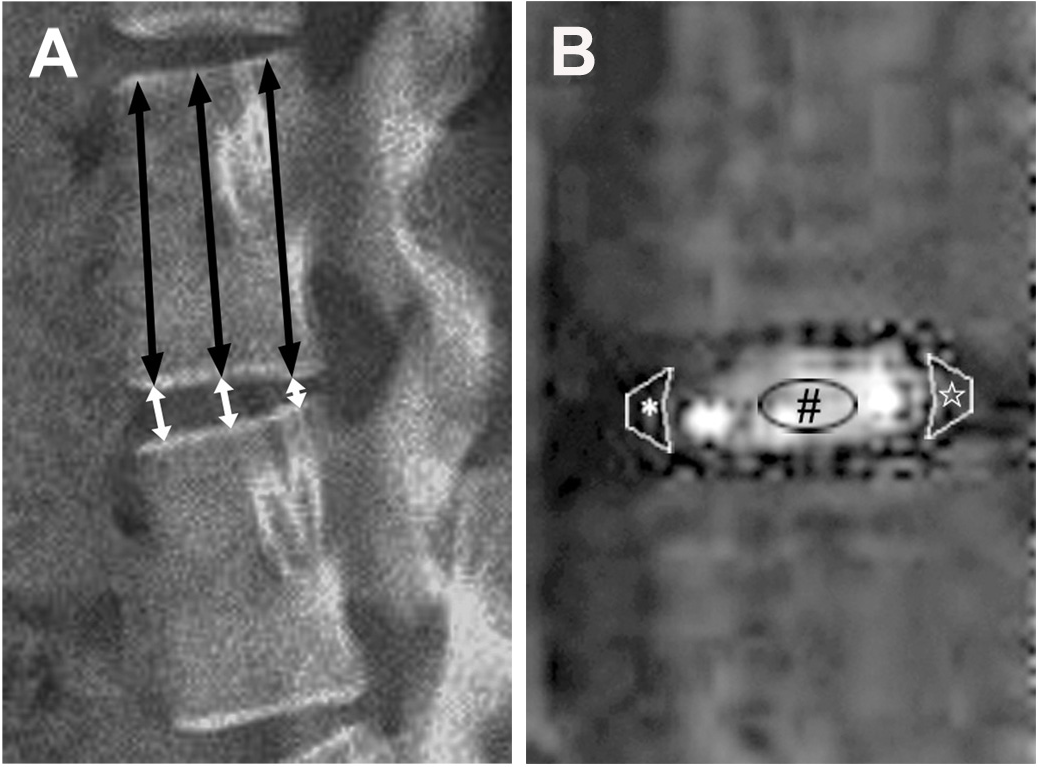

Supplement: Supplementary file 1 — Authors’ original file for figure 1 [file 12891_2014_2285_MOESM1_ESM.tiff]

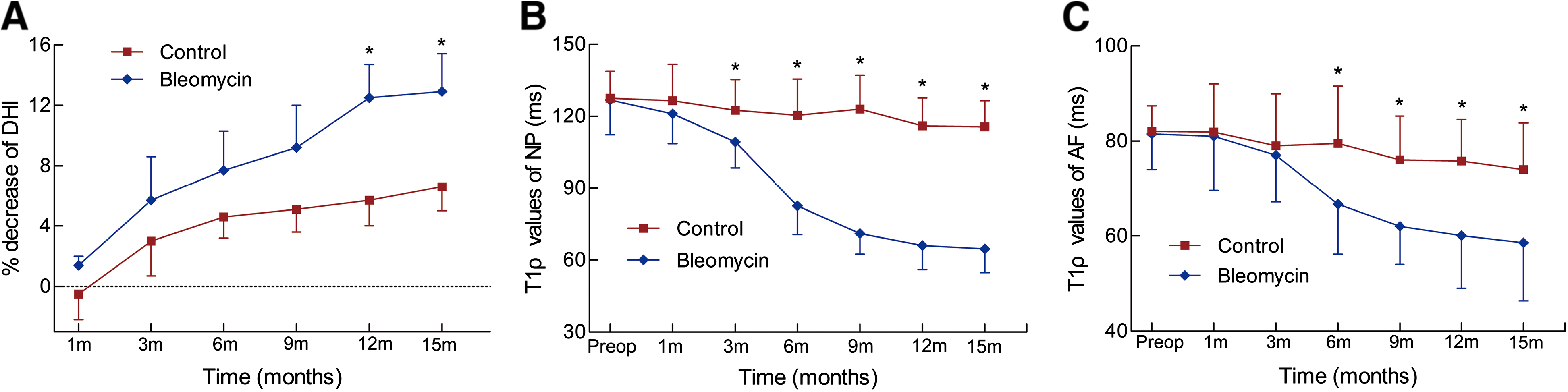

Supplement: Supplementary file 2 — Authors’ original file for figure 2 [file 12891_2014_2285_MOESM2_ESM.tiff]

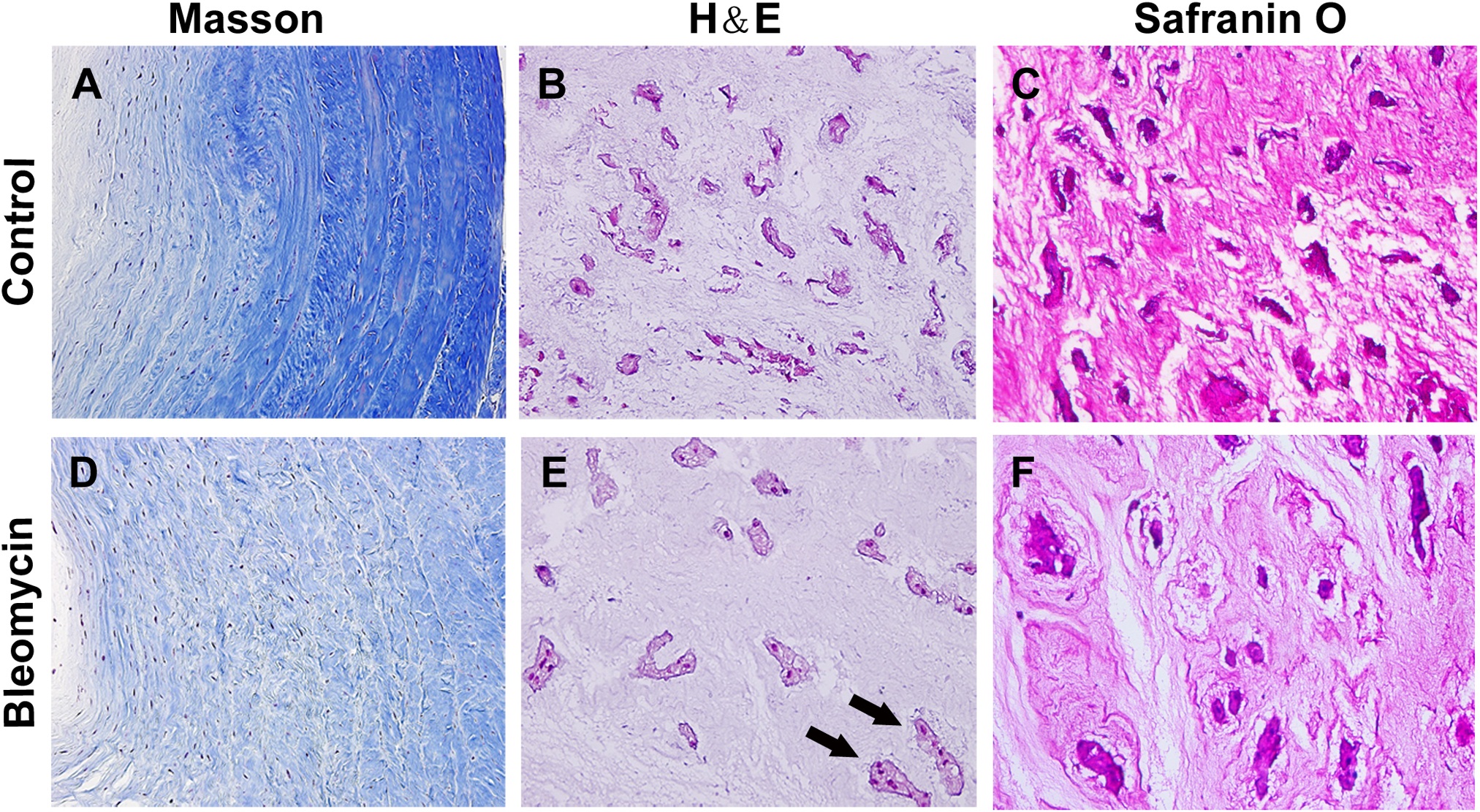

Supplement: Supplementary file 3 — Authors’ original file for figure 3 [file 12891_2014_2285_MOESM3_ESM.jpg]

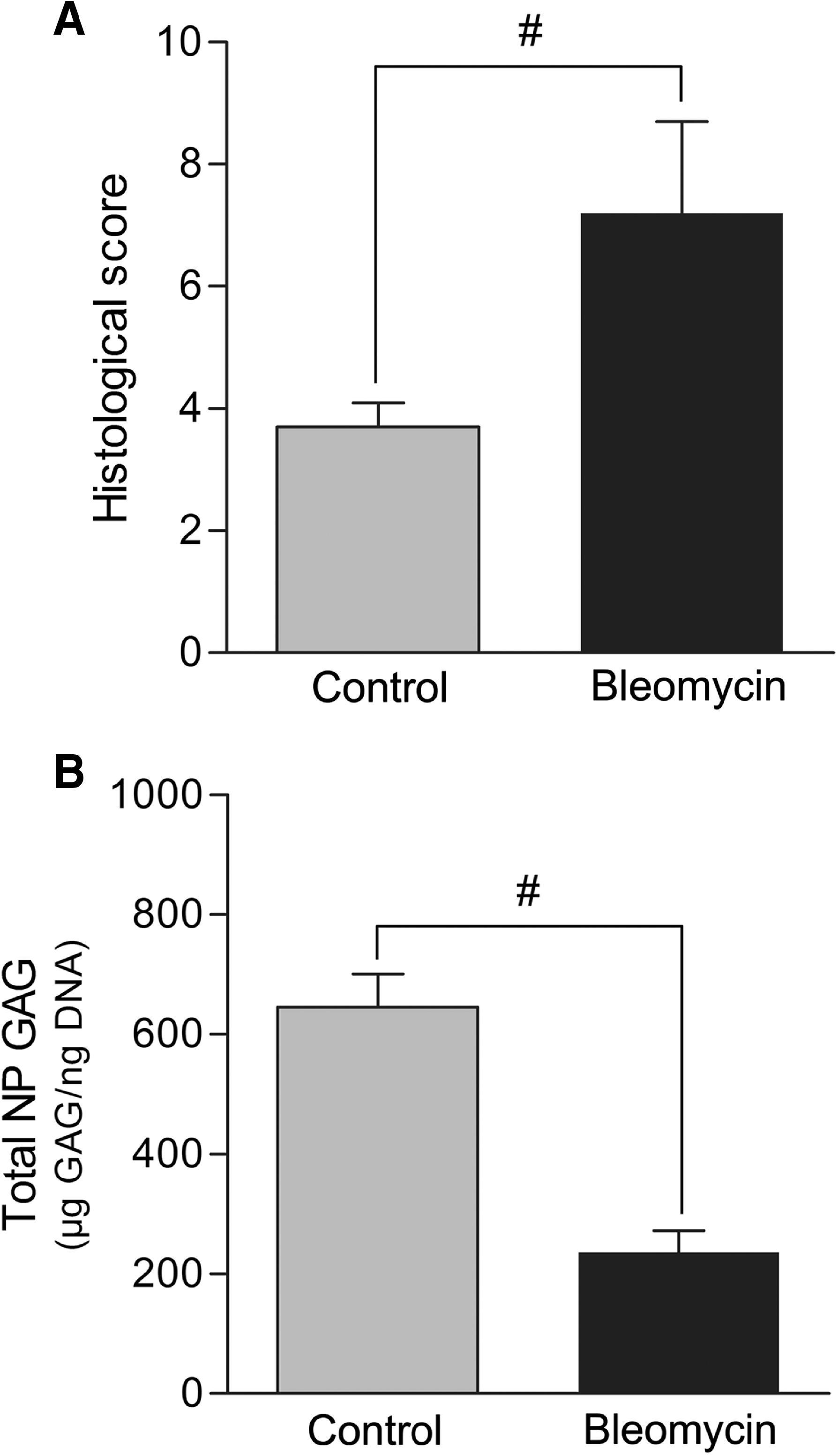

Supplement: Supplementary file 4 — Authors’ original file for figure 4 [file 12891_2014_2285_MOESM4_ESM.tiff]

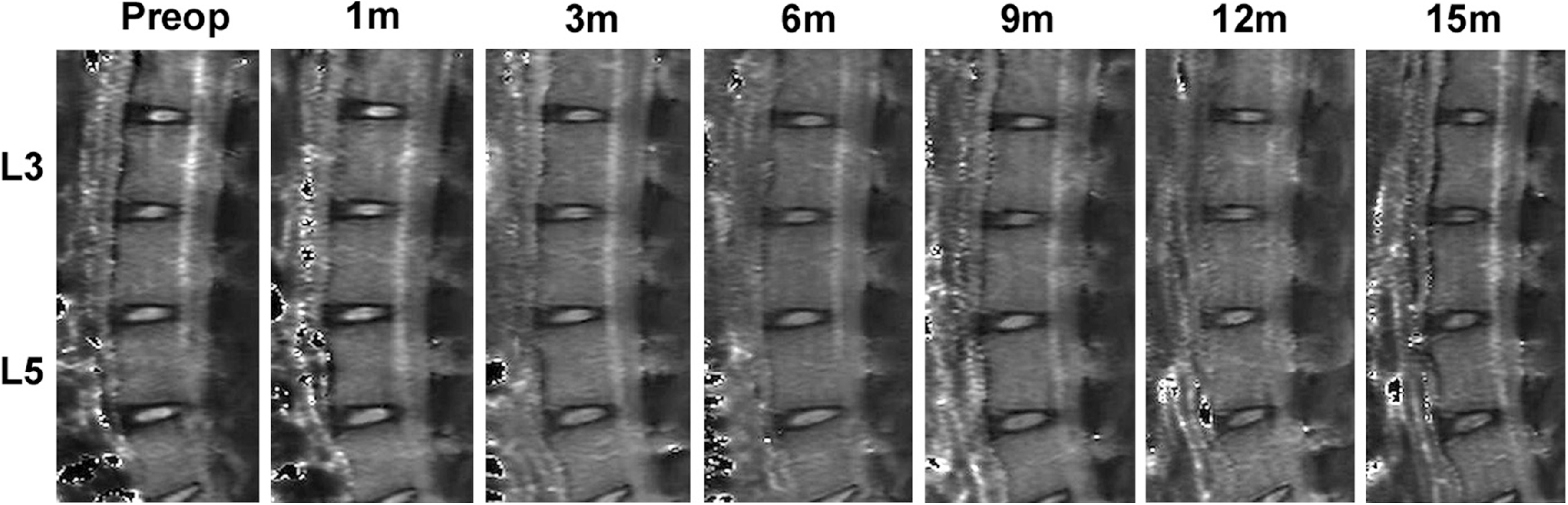

Supplement: Supplementary file 5 — Authors’ original file for figure 5 [file 12891_2014_2285_MOESM5_ESM.tiff]

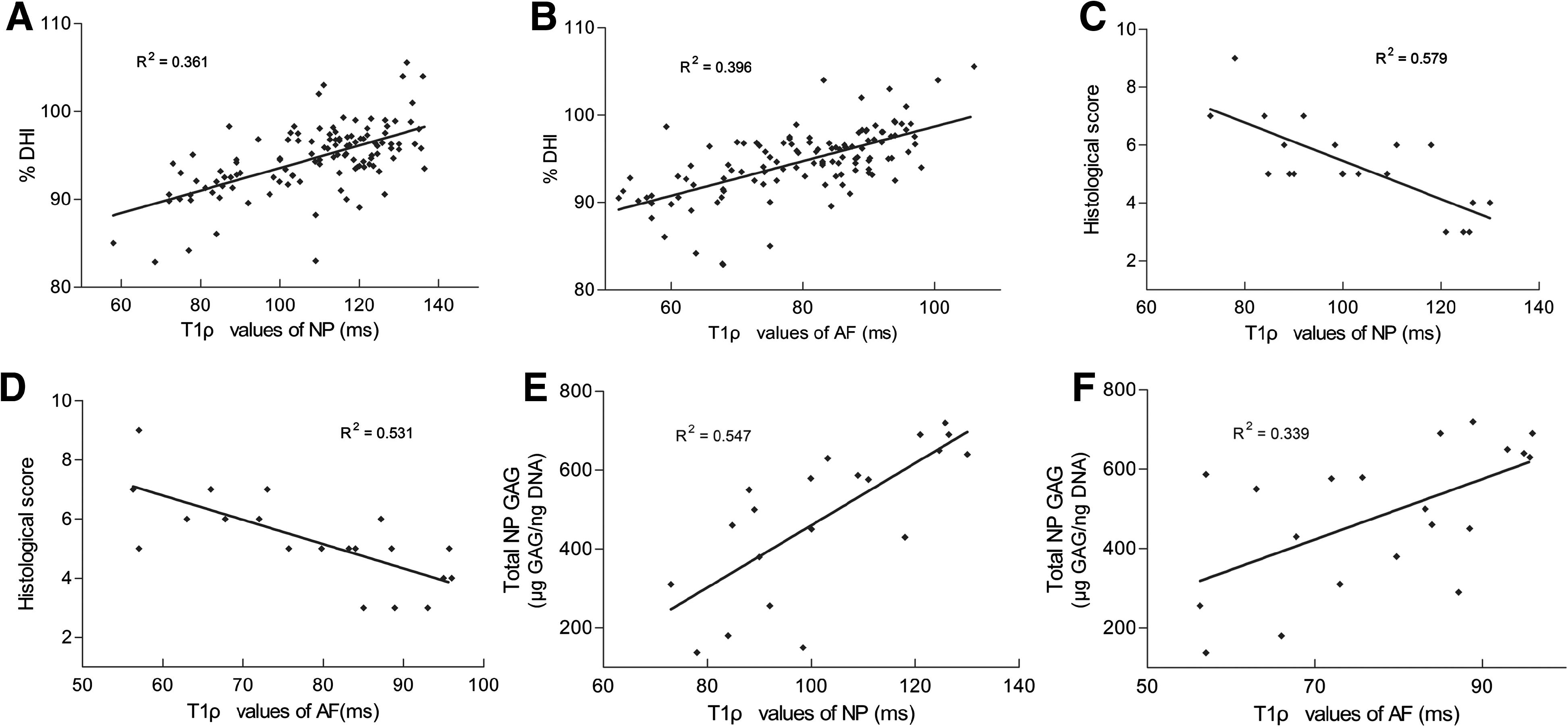

Supplement: Supplementary file 6 — Authors’ original file for figure 6 [file 12891_2014_2285_MOESM6_ESM.tiff]

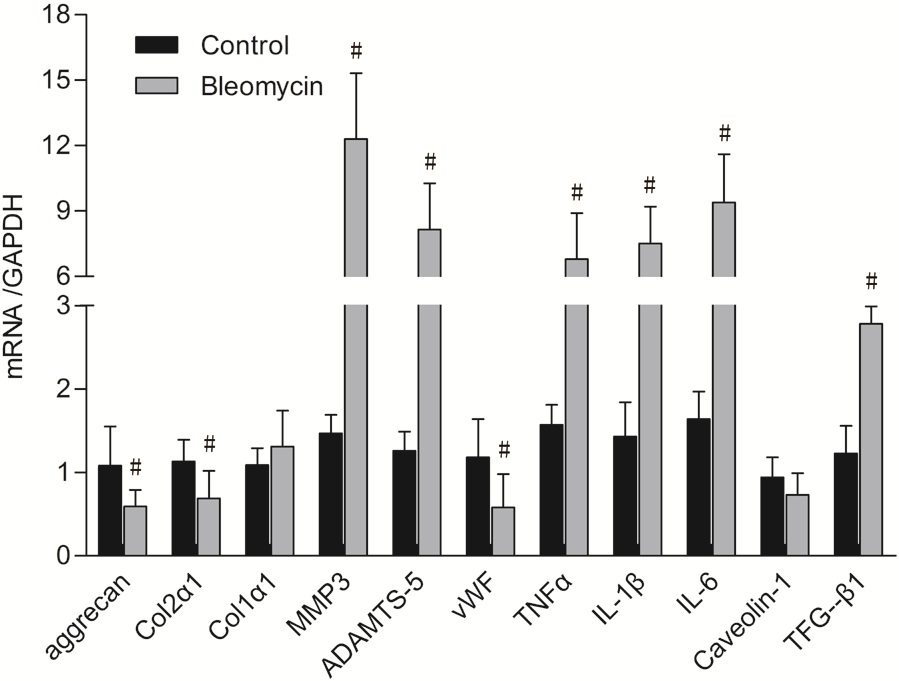

Supplement: Supplementary file 7 — Authors’ original file for figure 7 [file 12891_2014_2285_MOESM7_ESM.jpg]
